# Correction to “the Role of Hsa‐miR‐193a‐5p as an Important Factor for Control of Inositol in Alopecia Areata”

**DOI:** 10.1111/srt.70212

**Published:** 2025-07-31

AbdElneam AI, Al‐Dhubaibi MS, Bahaj SS, Mohammed GF, Atef LM. The role of hsa‐miR‐193a‐5p asan important factor for control of inositol in alopecia areata. *Skin Res Technol*. 2024;30(7):e13800. https://doi.org/10.1111/srt.13800.

Following the publication of the article, concerns were raised regarding the poorly defined scientific rationale and the lack of methodological details. This correction has been issued to address these concerns. The missing information, provided below, should be considered as an addition to the relevant sections of the published article.

**1.1 Aim of the study**

The selection of hsa‐miR‐193a‐5p in this study is based on prior research findings. Sheng and colleagues identified 22 microRNAs that were upregulated in severe AA, among which hsa‐miR‐193a‐5p was included [49]. Given the well‐established association between AA and autoimmune diseases, the involvement of hsa‐miR‐193a‐5p in various immune‐related disorders further supports its relevance. This microRNA has been implicated in conditions such as asthma [50], and multiple sclerosis (MS) [51], and it plays a regulatory role in innate immune cell responses [52]. Moreover, hsa‐miR‐193a‐5p has been shown to promote the secretion of key proinflammatory cytokines—including IL‐1β, IL‐6, IL‐8, and TNF‐α—through activation of the GSDMD axis, contributing to an inflammatory environment commonly observed in autoimmune conditions [53].

**2.2 RNA extraction and RT‐PCR**

Quantitative amplification was performed using specific primers for hsa‐miR‐193a‐5p were as follows: forward primer 5′‐TCTTTGCGGGCGAGATG‐3′ and reverse primer 5′‐GAACATGTCTGCGTATCTC‐3′ [54] and for the housekeeping control hsa‐miR‐16‐5p Forward Primer: 5′‐TAGCAGCACGTAAATATTGGCG‐3’ and Reverse Primer: 5′‐AACGCTTCACGAATTTG‐3′ (criteria of selection of housekeeping control is Stable Expression Across Samples, High Abundance, Minimal Interindividual Variation, Lack of Regulation in Many Conditions, and used previously in several  miRNA quantification) [55‐59]. Employing a Bio‐Rad iCycler (Bio‐Rad Laboratories, Hercules, CA). PCR cycling conditions consisted of 95°C for 2 min, followed by 40 amplification cycles of denaturation at 95°C for 15 s, annealing at 60°C for 30 s, and extension at 60°C for 30 s.

**5. DISCUSSION**

By focusing on the roles of the identified genes—SLC7A1, SRSF10, TNFRSF19, IPPK, and ZMYM4—our findings align with previous studies examining their involvement in AA and hair follicle biology. Zhang et al. identified 50 optimally upregulated genes associated with hair follicle regeneration, among which SLC7A1 was prominently listed [60]. Similarly, Youngquist and colleagues reported SLC7A1 as one of the genes associated with increased hair diameter [61]. Another study investigating androgenic alopecia found that SLC7A1, SLC7A5, and SLC7A11 were significantly downregulated in miniaturized hair follicles [62]. Furthermore, Xu et al. identified SLC7A11 as a potential biomarker for AA [63]. Both SLC7A1 and SLC7A11 belong to the SLC7 family, which is part of the broader solute carrier (SLC) superfamily involved in membrane transport processes.

The SRSF10 gene, a key regulator of alternative splicing [64, 65], may contribute to AA pathogenesis through its regulation of immune and stress response genes. Since AA is an autoimmune disorder marked by dysregulated immune signaling and hair follicle apoptosis [66], this connection is highly relevant. Several studies have demonstrated links between SRSF10 and immune regulation [67, 68].

TNFRSF19 has been directly associated with hair cycle regulation. Lim and colleagues identified TNFRSF19 as being involved in various biological processes such as the hair cycle, phase transitions, follicle development, maturation, and morphogenesis [69]. Additionally, Nakamura et al. observed that abnormalities in TNFRSF19 were associated with focal alopecia on the scalp and delayed development of secondary hair follicles [70].

Finally, both ZMYM4 and IPPK play crucial roles in the immune system, being essential for various immune functions [71, 72]. Their involvement supports a possible connection with AA, consistent with the autoimmune nature of the disease.

**References**

[49] Y. Sheng, S. Qi, R. Hu, et al., “Identification of Blood microRNA Alterations in Patients With Severe Active Alopecia Areata,” *Journal of Cellular Biochemistry* 120, no. 9 (2019): 14421–14430, https://doi.org/10.1002/jcb.28700.

[50] J. Tian, J. Ning, and Y Xu, “Bioinformatics Analysis of Differentially Expressed microRNAs in Children With Bronchial Asthma,” *Chinese Journal of Cellular and Molecular Immunology* 37, no. 10 (2021).

[51] N. Saeidi, H. Goudarzvand, H. Mohammadi, et al., “Dysregulation of miR‐193a Serves as a Potential Contributor to MS Pathogenesis via Affecting RhoA and Rock1,” *Multiple Sclerosis and Related Disorders* 69 (2023): 104468, https://doi.org/10.1016/j.msard.2022.104468.

[52] Z. Han, L. Li, H. Zhao, et al., “MicroRNA‐193a‐5p Rescues Ischemic Cerebral Injury by Restoring N2‐Like Neutrophil Subsets,” *Translational Stroke Research* 14, no. 4 (2023): 589‐607, https://doi.org/10.1007/s12975‐022‐01071‐y.

[53] J. Wang, X. Li, Y. Liu, et al., “CircHIPK3 Promotes Pyroptosis in Acinar Cells Through Regulation of the miR‐193a‐5p/GSDMD Axis,” *Frontiers in Medicine* 7 (2020): 88, https://doi.org/10.3389/fmed.2020.00088.

[54] A. B. Elmoselhi, M. Seif Allah, A. Bouzid, et al., “Circulating MicroRNAs as Potential Biomarkers of Early Vascular Damage in Vitamin D Deficiency, Obese, and Diabetic Patients,” *PLoS One* 18, no. 3 (2024): e0283608, https://doi.org/10.1371/journal.pone.0315309.

[55] H. J. Peltier, and G. J. Latham, “Normalization of MicroRNA Expression Levels in Quantitative RT‐PCR Assays: Identification of Suitable Reference RNA Targets in Normal and Cancerous Human Solid Tissues,” *RNA* 14, no. 5 (2008): 844‐852, https://doi.org/10.1261/rna.939908.

[56] l. Moldovan, K. E. Batte, J. Trgovcich, J. Wisler, C. B. Marsh, and M. Piper, “Methodological Challenges in Utilizing miRNAs as Circulating Biomarkers,” *Journal of Cellular and Molecular Medicine* 18, no. 3 (2014): 371‐390, https://doi.org/10.1111/jcmm.12236.

[57] E. M. Kroh, R. K. Parkin, P. S. Mitchell, and M. Tewari, “Analysis of Circulating microRNA Biomarkers in Plasma and Serum Using Quantitative Reverse Transcription‐PCR (qRT‐PCR),” *Methods* 50, no. 4 (2010): 298‐301, https://doi.org/10.1016/j.ymeth.2010.01.032.

[58] L. Wang, Y. Liu, L. Du, et al., “Identification and Validation of Reference Genes for the Detection of Serum MicroRNAs by Reverse Transcription‐Quantitative Polymerase Chain Reaction in Patients With Bladder Bancer,” *Molecular Medicine Reports* 12, no. 1 (2015): 615‐622, https://doi.org/10.3892/mmr.2015.3428.

[59] G. Zheng, H. Wang, X. Zhang, et al., “Identification and Validation of Reference Genes for qPCR Detection of Serum MicroRNAs in Colorectal Adenocarcinoma Patients,” *PLoS One* 8, no. 12 (2013): e83025, https://doi.org/10.1371/journal.pone.0083025.

[60] C. Zhang, Y. Li, J. Qin, C. Yu, G. Ma, H. Chen, and X. Xu. “TMT‐Based Quantitative Proteomic Analysis Reveals the Effect of Bone Marrow Derived Mesenchymal Stem Cell on Hair Follicle Regeneration,” *Frontiers in Pharmacology* 12 (2021): 658040, https://doi.org/10.3389/fphar.2021.658040.

[61] R. S. Youngquist, J. Xu, R. L. Binder, et al., “Systems and Methods for Identifying Cosmetic Agents for Hair/Scalp Care Compositions,” *Google Patents* (2018).

[62] J. Li, X. Duan, F. Cheng, G. Li, and Z. L. Deng, “Impaired Arginine Metabolism in Hair Follicles: A Potential Mechanism in Androgenetic Alopecia,” *Research Square* (2023), https://doi.org/10.21203/rs.3.rs‐3629594/v1.

[63] W. Xu, D. Wei, and X. Song, “Identification of SLC40A1, LCN2, CREB5, and SLC7A11 as Ferroptosis‐Related Biomarkers in Alopecia Areata Through Machine Learning,” *Scientific Reports* 14, no. 1 (2024): 3800, https://doi.org/10.1038/s41598‐024‐54278‐4.

[64] L. Shkreta, A. Delannoy, A. Salvetti, and B. Chabot, “SRSF10: an Atypical Splicing Regulator With Critical Roles in Stress Response, Organ Development, and Viral Replication,” *RNA* 27, no. 11 (2021): 1302‐1317, https://doi.org/10.1261/rna.078879.121.

[65] L. Shkreta, J. Toutant, M. Durand, J. L. Manley, and B. Chabot, “SRSF10 Connects DNA Damage to the Alternative Splicing of Transcripts Encoding Apoptosis, Cell‐Cycle Control, and DNA Repair Factors,” *Cell Reports* 17, no. 8 (2016): 1990‐2003, https://doi.org/10.1016/j.celrep.2016.10.071.

[66] A. Gilhar, A. Etzioni, and R. Paus, “Alopecia areata,” *The New England Journal of Medicine* 366, no. 16 (2012), https://doi.org/10.1056/NEJMra1103442.

[67] X. Luo, Z. Zhang, S. Li, et al., “SRSF10 Facilitates HCC Growth and Metastasis by Suppressing CD8(+)T Cell Infiltration and Targeting SRSF10 Enhances Anti‐PD‐L1 Therapy,” *International Immunopharmacology* 127, (2024): 111376, https://doi.org/10.1016/j.intimp.2023.111376.

[68] J. Cai, L. Song, F. Zhang, et al., “Targeting SRSF10 Might Inhibit M2 Macrophage Polarization and Potentiate Anti‐PD‐1 Therapy in Hepatocellular Carcinoma,” *Cancer Communications* 44, no. 11 (2024): 1231‐1260, https://doi.org/10.1002/cac2.12607.

[69] Y. C. Lim, H. Kim, S. M. Lim, and J. S. Kim, “Genetic Analysis of a Novel Antioxidant Multi‐Target Iron Chelator, M30 Protecting Against Chemotherapy‐Induced Alopecia in Mice,” *BMC Cancer* 19, no. 1 (2019):149, https://doi.org/10.1186/s12885‐019‐5323‐z.

[70] M. Nakamura, M. R. Schneider, R. Schmidt‐Ullrich, and R. Paus, “Mutant Laboratory Mice With Abnormalities in Hair Follicle Morphogenesis, Cycling, and/or Structure: an update,” *Journal of Dermatological Science*. 69, no. 1 (2013): 6‐29, https://doi.org/10.1016/j.jdermsci.2012.10.001.

[71] H. Cibis, A. Biyanee, W. Dörner, H. D. Mootz, and K. H. Klempnauer, “Characterization of the Zinc Finger Proteins ZMYM2 and ZMYM4 as Novel B‐MYB Binding Proteins,” *Scientific Reports* 10, no. 1 (2020): 8390, https://doi.org/10.1038/s41598‐020‐65443‐w.

[72] N. K. Pulloor, S. Nair, K. McCaffrey, et al., “Human Genome‐Wide RNAi Screen Identifies an Essential Role for Inositol Pyrophosphates in Type‐I Interferon Response,” *PLoS Pathogens* 10, no 2 (2014): e1003981, https://doi.org/10.1371/journal.ppat.1003981.

